# Effects of *Tagetes erecta* on the Pigmentation of Large Yellow Croaker(*Larimichthys crocea*)

**DOI:** 10.1155/anu/3616724

**Published:** 2025-10-08

**Authors:** Siyu Zhang, Tianwei Wang, Lin Chen, Chengwei Huang, Yali Wang, Rongrong Ma, Jianping Wang, Zehui Su, Subin Cui, Dongmin Zhu, Jiahui Zhu, Kun Hu

**Affiliations:** ^1^National Demonstration Center for Experimental Fisheries Science Education, Shanghai Ocean University, Shanghai 201306, China; ^2^National Pathogen Collection Center for Aquatic Animals, Shanghai Ocean University, Shanghai 201306, China; ^3^Key Laboratory of Freshwater Aquatic Genetic Resources, Ministry of Agriculture, Shanghai Ocean University, Shanghai 201306, China; ^4^State Key Laboratory of Mariculture Biobreeding and Sustainable Goods, Yellow Sea Fisheries Research Institute, Chinese Academic of Fishery Sciences, Qingdao 266071, Shandong, China; ^5^Xiangshan Fishery Bureau, Ningbo 315708, Zhejiang, China; ^6^Ningbo Academy of Oceanology and Fisheries, Ningbo 315012, China; ^7^School of Marine Sciences, Ningbo University, Ningbo 315211, Zhejiang, China; ^8^Ningbo Yonggang Aquatic Seed Technology Co., Ltd., Ningbo 315000, China

**Keywords:** *Amaranthus* spp., astaxanthin, body color, *Larimichthys crocea*, *Tagetes erecta L*.

## Abstract

*Larimichthys crocea* (*L. crocea*), an important species in mariculture, is highly valued for its nutritional benefits, delicate flavor, and distinctive golden body, making it a popular choice among consumers. However, suboptimal breeding conditions and high stocking densities, often lead to reduced flesh texture and a deterioration in body color. Given the significant role body color plays in determining the commercial value of this species, this study evaluated the impact of various dietary supplements on color enhancement. One basal diet and three separate experimental diets were formulated: one supplemented with 200 mg/kg *Tagetes erecta* extract (2% lutein), one with 200 mg/kg astaxanthin (2%), and one with 200 mg/kg amaranth (*Amaranthus* spp.) leaf extract (85%). The results indicated that *T. erecta* was more effective in enhancing the body color of *L. crocea* than the other two supplements after 28 days of feeding, with the *a*^*∗*^ value (red–green) 1.25-fold higher than the control group and ventral *b*^*∗*^ value (yellow–blue) was significantly elevated (*p* = 0.028) compared to the control group. Initial mechanistic studies revealed that *T. erecta* enhanced body color through regulating carotenoid uptake and melanogenesis. These findings provide valuable insights for optimizing feeding strategies to improve the market appeal and commercial value of *L. crocea* in mariculture.

## 1. Introduction

*Larimichthys crocea* (*L. crocea*), as a key marine aquaculture organism in China, holds the top position in annual mariculture yield [1, 2]. It is highly prized for its rich nutritional content, including protein, vitamins, and trace elements, as well as its tender meat and pleasing taste [3]. Owing to the declining availability of wild population resources, artificial breeding has become increasingly prevalent in recent years. Despite this, cultured *L. crocea* faces challenges such as diminished skin pigmentation, degraded muscle flavor, and a looser meat texture compared to wild specimens [4–6]. Body color is an essential indicator of both the health and market value of fish and it plays a crucial role in classification within the industry. Thus, maintaining or enhancing the color characteristics of *L. crocea* during artificial breeding is vital to improving its economic value.

Fish body coloration is composed of chromatophores and carotenoids serve as the primary color-presenting substances in erythrophores and xanthophores. The formation of chromatophores relies on gene-governed neural crest-derived precursor cells [7]. Fish are unable to synthesize carotenoids de novo, so their pigmentation primarily depends on carotenoids in their diet [8]. Studies have shown that supplementing with carotenoids can enhance the body color of fish [9]. Common carotenoids found in fish include β-carotene, lutein, zeaxanthin, tunaxanthin, and astaxanthin. Regarding body color enhancement, Yi et al. [10] found that the body color of *L. crocea* is largely influenced by the deposition of yellow pigments. Supplementing the diet of juvenile *L. crocea* with astaxanthin and lutein for 9 weeks significantly increased the red and yellow values of their skin. Similarly, Yi et al. [11] demonstrated that incorporating shrimp shell powder into the feed improved epidermal pigmentation while elevating carotenoid compound concentrations in *L. crocea*.

However, there remains a gap in understanding the effects of natural plant-extract pigment supplements on body color in *L. crocea*. This study selected three widely distributed, highly safe, and carotenoid-rich plant extracts, to explore the effects of these three kinds of pigment supplements on the body color formation of *L. crocea*.

*Tagetes erecta L*., a plant rich in natural lutein, has been widely used as a coloring agent in food products and as a nutritional component in diets for both humans and livestock. The petals of *T. erecta* are a major source of lutein for the food and pharmaceutical industries [12]. In zebrafish (*Danio rerio*), bioactive components derived from *T. erecta* interact with the melanocortin-1 receptor, potentially inhibiting melanin biosynthesis [13]. However, the effects of marigold (*T. erecta*) on skin coloration in *L. crocea* have not been systematically investigated. Another source of natural pigments, *Amaranthus tricolor*, is recognized for its high antioxidant content, pigments, minerals, and phytochemicals [14]. In the Chinese feed additive variety list (2013), amaranth is a colorant that can be used in pets and ornamental fish. Known for its nontoxicity, bright color, and stable nature, it is frequently used as a safe and nutritious natural edible pigment [15]. Astaxanthin, a well-known carotenoid, is commonly included in fish feed to enhance red pigmentation and improve the overall body color of fish.

Consequently, the present study explored the impact of three distinct experimental diets, respectively, on the apparent color change and underlying mechanisms in *L. crocea*, with the aim of providing a reference for improving both color and production in artificial breeding systems. This study conducted a comparative assessment of the differential impacts on pigmentation in *L. crocea* among three separate dietary supplements: *T*.*erecta*, amaranth (*Amaranthus* spp.) leaf extract, and astaxanthin—a pigment commonly used in aquaculture.

## 2. Materials and Methods

The experimental procedures involving animal subjects and the research methodology underwent rigorous evaluation and received formal approval by the Ethics Committee of Shanghai Ocean University (Approval Number: SHOU-DW-2023-17).

### 2.1. Fish Management and Experimental Design

The breeding site was located at the cage base of Baishi Mountain, Xiangshan Port, Ningbo Institute of Ocean and Fisheries Research, Zhejiang Province. Healthy “Yongdai No.1” *L. crocea* fry were selected and placed in a seawater cage (3.0 m × 3.0 m × 4.0 m) for 2 weeks, during which they were fed with pellet feed provided by Zhejiang Qiangpu Biotechnology Co., Ltd. prior to the formal experiment, the commercial feed contained 11% moisture, 46% crude protein, and 2% crude fat. After the 2-week temporary feeding period, the experimental fish were starved for 24 h. About 30–40 g *L. crocea* juveniles with strong physiques and consistent specifications were randomly allocated into experimental groups, with each enclosure containing a cohort of 150 individuals. To explore the effect of feeding natural pigments on the body color of *L. crocea* during the breeding process, three experimental groups were setup: *T. erecta* extract addition group (LT), astaxanthin addition group (XT), and amaranth addition group (AT). Each treatment was replicated three times. *Tagetes erecta* extract standardized to contain 2% (*w*/*w*) lutein as constituent and astaxanthin (1%, *w*/*w*) were purchased from Shanxi Huike Plant Development Co., Ltd., and 85% (*w*/*w*) amaranth leaf extract was purchased from Rouya (Shanghai) Food Additives Trade Co., Ltd. Based on preliminary experimental results from our laboratory and previous studies by Swian et al. [16], Biswas et al. [17], and Pailan et al. [18], dietary supplementation with 80–400 mg/kg lutein or astaxanthin extract for 4 weeks significantly improved fish body coloration. Three experimental groups were added pigment supplements: *T. erecta* extract 200 mg/kg, astaxanthin 200 mg/kg, and amaranth leaf extract 200 mg/kg. The control group was fed a basal diet without pigment supplement. During the breeding process, the pigment supplements were weighed daily and mixed with water and adhesive (purchased from Jiangsu Fishery Doctor Aquaculture Technology Co., Ltd.). The adhesive exhibits chemical inertness toward pigment additives and feed components. All experimental groups were fed the same basal diet. The growth experiment employed satiation feeding, with the first feeding at 5:00 AM and the second feeding at 4:30 PM. The formal experimental breeding cycle lasted 4 weeks. During the experiment, the water temperature ranged from 21.0 to 24.5°C, pH levels were maintained between 8.0 and 8.2, dissolved oxygen concentrations varied from 8.2 to 8.9 mg/L, and the salinity was approximately 27.9‰.

### 2.2. Sampling

After 28 days of culture, the test fish were subjected to a 24-h starvation period, followed by sample collection. The ventral skin of *L. crocea* exhibits a silvery-white coloration under light conditions, but turns golden-yellow when maintained in darkness [19]. Preliminary tests confirmed that the ventral skin coloration of *L. crocea* stabilizes after 40 min of dark exposure. Consequently, the collection procedure was divided into light and dark treatment groups. Seven individuals from each experimental enclosure were randomly chosen and anesthetized using a eugenol solution at a dilution ratio of 1:10,000. Data on weight, body length, and body height were recorded. Growth parameters were measured as follows:

  $$\begin{array}{c} \text{Percent weight gain }\left(\text{PWG};\;\%\right)=100\times \left(Wt-W_{0}\right)/W_{0}. \end{array}$$

  $$\begin{array}{c} \text{Specific growth ratio }\left(\text{SGR};\;\%/\text{day}\right)=100\times \left(\text{Ln }Wt-\text{Ln }W_{0}\right)/t. \end{array}$$

*W*_*t*_ and *W*_0_ denote final body weight (FBW) and initial body weight (g), respectively, and *t* represents the culture period (days).

The light treatment group was sampled under natural light, while the dark treatment group was first acclimated in a dark room for 40 min before sampling. Continuous oxygen delivery in the dark treatment group ensured the activity of the fish. *L*^⁣^*∗*^^, *a*^*∗*^, and *b*^*∗*^ values for ventral regions were measured. Additionally, 1 cm × 2 cm skin samples were taken from the control, AT, and LT groups under dark treatment conditions. These samples were placed in liquid nitrogen and preserved in ultralow temperature storage units maintained at −80°C. The skin pigment content in both the control and LT groups was measured and expression changes of differentially expressed genes (DEGs) and DEMs between the control and LT groups were assessed.

### 2.3. Body Color Determination

A portable spectrophotometer (CS-410, Chromatograph Technology, Zhejiang Co., Ltd.) was used to measure skin brightness (*L*^⁣^*∗*^^), red–green (*a*^*∗*^), and yellow–blue (*b*^*∗*^) at ventral skin under both light (L) and dark (D) conditions. The average values for each measurement were calculated. These values were then used to compute five color coefficients: *a*^*∗*^/*b*^*∗*^, (*a*^*∗*^/*b*^*∗*^)2, chroma value (CV), and hue value (HV), according to the following calculation formulas:

  $$\begin{array}{c} \text{CV}={\left(a^{*2}+b{\text{⁣}}^{*2}\right)}^{1/2}. \end{array}$$

  $$\begin{array}{c} \text{HV}=\text{arctan}\left(b^{*}/a^{*}\right),\left(a^{*}>0,b^{*}>0\right)\text{ or }180{}^{\circ}+\text{arctan}\left(b^{*}/a^{*}\right),\left(a^{*}<0,b^{*}>0\right). \end{array}$$

### 2.4. Transcriptome Analysis

#### 2.4.1. RNA Extraction

Total RNA from control group and LT group were performed utilizing TRlzol Reagent (Invitrogen, CA, USA). The purity and concentration of the extracted RNA were quantified with the NanoDrop 2000 spectrophotometer (Thermo Scientific, USA), and the integrity of the RNA was verified using the Agilent 2100 Bioanalyzer (Agilent Technologies, Santa Clara, CA, USA)

#### 2.4.2. Preparation of Sequencing Libraries and Assembling Transcriptomes

For the construction of RNA sequencing (RNA-seq) libraries, high-quality RNA samples (3 μg) were processed for RNA-seq library preparation. mRNA enrichment was achieved by capturing polyadenylated transcripts using magnetic beads conjugated with poly-T oligonucleotides. Construction of the RNA-seq libraries was carried out with the VAHTS Universal V6 RNA-seq kit, adhering to the standard operating procedures provided by the manufacturer. cDNA fragments within the size range of 400–500 bp were purified using the AMPure XP system (Beverly, California, USA). The Illumina PCR Primer Cocktail was utilized to amplify adapter-ligated DNA fragments through 15 rounds of PCR. Postpurification with the AMPure XP system, the library concentration was measured using the Agilent 2100 Bioanalyzer with a high-sensitivity DNA chip. The final libraries were sequenced using the Illumina NovaSeq 6000 system [20].

#### 2.4.3. Transcriptome Sequencing

High-throughput sequencing was conducted on the NovaSeq 6000 platform (Illumina), generating bidirectional sequence fragments of 150 bp read length. Using the fastp software tool to subject quality control (QC) and preprocessing [21]. After filtering out substandard reads, the resulting high-quality data were aligned against the reference genome using the HISAT2 software for sequence mapping [22]. The level of gene expression was determined through the FPKM method [23] and read counts were generated through the application of the HTSeq-count software tool [24]. *Q* value < 0.05 and foldchange > 2 or foldchange < 0.5 identified using DESeq2 were considered as DEGs. Hierarchical clustering analysis of DEGs was implemented in R (version 3.2.0) to examine expression trends across distinct groups and samples. Furthermore, a radar plot depicting the top 30 DEGs was constructed using the ggradar, highlighting both upregulated and downregulated gene expression patterns.

#### 2.4.4. Gene Ontology (GO) and Kyoto Encyclopedia of Genes and Genomes (KEGG) Pathway Enrichment Analyses

GO [25] and KEGG [26] pathway enrichment analysis of DEGs was carried out using the Reactome and WikiPathways resources, with the hypergeometric test applied for statistical evaluation in R (version 3.2.0). Significant terms were identified for each analysis. KEGG pathway analysis was conducted using Cluster Profiler (v 3.4.4) software to examine the functional pathways associated with the DEGs.

### 2.5. Quantitative Reverse Transcription PCR (qRT-PCR) Verification

To validate the RNA-Seq results, five genes were selected for a qRT-PCR analysis using a 2 × SYBR GREEN Master Mix. Primers were developed using sequence data obtained from the NCBI database and β-actin served as the internal control gene for normalization purposes. The thermal cycling conditions were as follows: 95°C for 10 min and 40 cycles at 95°C for 10 s and 60°C for 30 s. A melting curve was generated at the end of the amplification process. Primers for RT-PCR are shown in Table 1.

### 2.6. Metabolome Analysis

#### 2.6.1. Sample Extraction

Reagents were precooled at −20 °C prior to the experiment. Samples from the control group and the LT group (30 mg) were weighed into EP tube and add two small steel balls. Then, 400 μL of a 4:1 (*v*/*v*) methanol–water mixture, which included a mixed internal standard at 4 μg/mL, was introduced. Precooling the mixture in a −40°C refrigerator for 2 min, the samples were ground using an automatic rapid grinding instrument (Wonbio-E, Shanghai Wanbai Biotechnology Co., Ltd.) at 60 Hz. The extraction process was carried out ultrasonically in an ice-water bath, followed by incubation at −40 °C for 2 h. The samples were then subjected to centrifugation at 13,000 rpm for 20 min at 4°C. A 150 μL aliquot was withdrawn using a syringe, passed through a 0.22 μm organic membrane filter, introduced into an LC via. Processed samples were preserved at −80 °C pending liquid chromatography/mass spectrometry (LC-MS) analysis. For quality assurance, a pooled QC sample was generated by combining equal volumes of extracts from each individual sample.

#### 2.6.2. LS-MS Analysis

The samples were analyzed on an integrated LC-MS platform comprising a Waters ACQUITY UPLC I-Class Plus and a Thermo QE Plus high-resolution tandem mass spectrometer.

##### 2.6.2.1. LC Conditions

The chromatographic column used was the ACQUITY UPLC HSS T3 (100 mm × 2.1 mm, 1.8 μm); column temperature: 45°C; injection volume: 3 μL; flow rate: 0.35 mL/min. The mobile phases consisted of A (water containing 0.1% formic acid) and B (acetonitrile). The gradient elution procedure is detailed in Table 2.

##### 2.6.2.2. Mass Spectrometer Conditions

After T3 chromatography separation, primary and secondary spectra were acquired using the mass spectrometer.

### 2.7. Targeted Quantitative Detection of Lutein and Astaxanthin

#### 2.7.1. Sample Pretreatment

Biological samples stored at −80 °C were ground to a fine powder using a low-temperature grinder (50 Hz, 1 min) in a liquid nitrogen environment. Approximately 100 mg of the ground sample (±5 mg) was weighed, mixed with 1.5 mL of acetone/n-hexane/ethanol (1:1:2, *V*/*V*/*V*) extraction solvent, and vortexed thoroughly. The extract was sonicated for 10 min using an ultrasonic device (KQ5200E, Kunshan Shumei Ultrasonic Instrument Co., Ltd.), then, incubated at 4°C for 16 h. The shaken sample solution was centrifuged at 12,000 rpm for 5 min. The supernatant was transferred to a new centrifuge tube and concentrated in a low-temperature cold trap (Auto R1-Plus, Beijing Jiam Instrument Co., Ltd.). After concentration, the residue was reconstituted in 100 μL of methanol/tert-butyl methyl ether (3:1, *V*/*V*), subjected to filtration using a 0.22 μm membrane, following which the filtrate was transferred into an injection vial.

#### 2.7.2. LC-MS Conditions

##### 2.7.2.1. LC Conditions

Chromatographic conditions: YMC Carotenoid C30 column (100 ×, 4.6 mm, 5 μm); temperature: 35 °C, volume of injection: 1 μL; flow rate: 0.3 mL/min; Phase A: 0.1% formic acid, Phase B: methanol; elution gradient: 0–2 min, A phase = 90%; 2–6 min, A phase = 90%–10%; 6–8 min, A phase = 10%; 8–8.1 min, A phase = 10%–90%; 8.1–10 min, A phase = 90%.

##### 2.7.2.2. Mass Spectrometer Conditions

Ion source: ESI (Turbo Spray); polarity: positive; spray voltage: 5500 V; sheath gas: 30 psi; collision gas pressure: 9 psi; atomization temperature: 550°C; scanning mode: MRM. Targeted detection of compound ion pairs and collision voltage were shown in Table 3.

##### 2.7.2.3. QC

In order to minimize the impact of instrument stability on test results, a quality assurance protocol was implemented. A QC sample was introduced following the analysis of 10 experimental samples. The total ion chromatogram (TIC) for the MS analysis of the control sample was overlaid to ensure data repeatability and reliability.

##### 2.7.2.4. Standard Curve and Content Calculation

Standard solutions of varying concentrations were systematically prepared for method validation, and the ion abundance values corresponding to the target quantification ions of each concentration were measured and documented. Standard curves for different substances were generated by plotting the standard peak area against the standard concentration. The integral peak area ratios from all analyzed specimens were processed using the linear regression equation of the standard curve to determine the corresponding analyte concentrations. Further substitution into the formula yielded the substance content data for the actual samples. The content calculation formula is as follows:

  $$\begin{array}{c} \text{Content (ng/g)}=c\times V/m. \end{array}$$

*c*: The concentration value (ng/μL) applying the measured peak area ratio of the sample to the standard curve.

*V*: Volume (μL) of the sample reconstitution.

*m*: Weighed sample mass (g).

### 2.8. Statistical Analysis

All data were compiled and processed statistically using Microsoft Excel 2016. Prior to statistical analyses, normality was assessed using the Shapiro–Wilk test and homogeneity of variance evaluated via Levene's test. Growth performance, skin coloration, gene expression quantification, and targeted metabolomics data were analyzed by one-way ANOVA, with statistical significance set at *p* < 0.05. Results are presented as mean ± standard error of the mean (SEM).

## 3. Results

### 3.1. Changes in Body Color of Experimental Fish

Table 4 presents the growth performance of *L. crocea* fed diets supplemented with pigment additives for 28 days. Fish receiving pigment-enriched feeds showed no significant changes in growth performance compared to the control group. FBW, percent weight gain (PWG), and specific growth rate (SGR) remained statistically comparable (*p* > 0.05) between pigment-supplemented and basal diet-fed cohorts.

**Figure 1:**
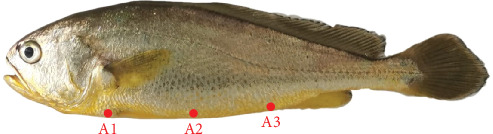
Color value measurement position of *Larimichthys crocea*. Reference to Chinese Fishery Standard SC/T 3123-2022.

**Figure 2:**
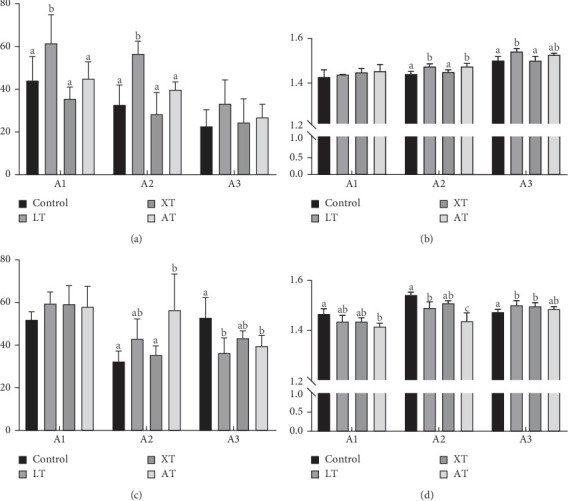
Chroma value (CV) and hue value (HV) of the ventral skin in different treatment groups. Bar graphs represent the means and standard error means of four biological replicates of values for each skin measurement site. Different superscript letters at the same sampling position indicate statistically significant differences (*p* < 0.05). (A) CV values of Points A1, A2, and A3 in each group under natural light conditions. (B) HV values of Points A1, A2, and A3 in each group under natural light conditions. (C) CV values of Points A1, A2, and A3 in the dark sampling group. (D) HV values of Points A1, A2, and A3 in each group under dark conditions. Number of replicates is 4.

After the experiment, the ventral skin of the control group and three experimental groups were measured using a spectrophotometer under both light and dark conditions. The measurement positions are depicted in Figure 1. The CV and HV were subsequently calculated. The results are presented in Table 5 and Figure 2. Under dark conditions, the chroma of LT and AT increased at Locations A2 and A3. All three experimental groups exhibited HV to 0° (indicating redder color) at Locations A1 and A2 under dark conditions, with LT and AT showing similar trends in CV and HV changes. The XT group displayed minimal changes. Under light conditions, only LT group showed significantly higher chroma than the control group at Locations A1 and A2.

### 3.2. Illumina Sequencing and Quality Assessment

After count data were obtained, protein-coding genes were filtered to exclude those with zero counts across all samples. A total of 21,355 genes were detected, including 19,210, 19,358, 19,593, and 19,102 genes in control group. The LT group samples exhibited 19,744, 19,503, 19,056, and 18,853 detected genes, respectively. The number of genes detected in the two groups converged.

**Figure 3:**
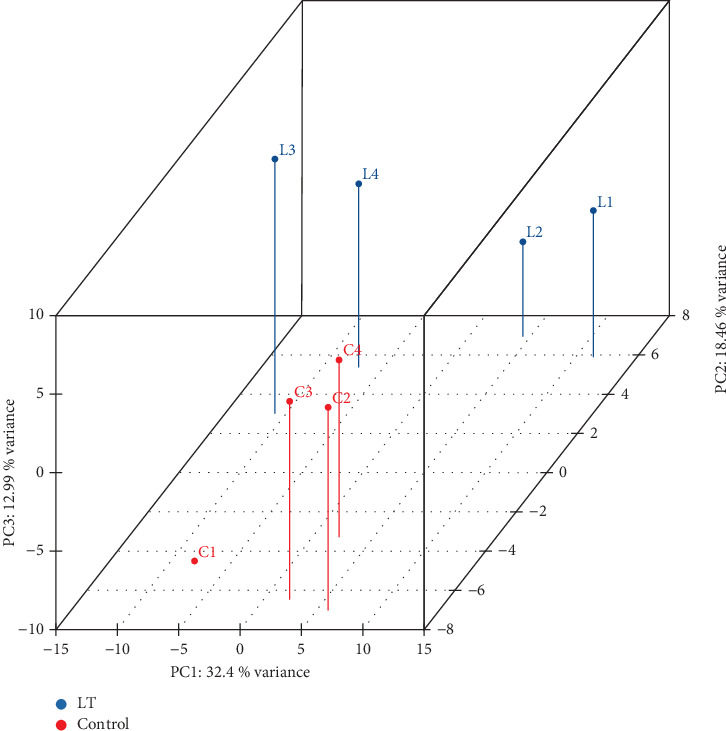
PCA structure of differentially expressed genes. PCA performs dimensionality reduction through orthogonal linear transformation, projecting data onto a new set of axes termed principal components. These components are ordered by descending eigenvalues of the covariance matrix, which correspond to their variance magnitudes. Specifically, the first principal component (PC1) represents the direction of maximum variance in the original data space. PCA revealed clear segregation between lutein-supplemented (LT) and control groups along PC1 (32% variance), indicating variation in samples induced by pigment supplement.

Principal component analysis (PCA) was conducted to assess the spatial arrangement and clustering characteristics of samples across different groups. The results are shown in Figure 3.

### 3.3. Analysis of DEGs

**Figure 4:**
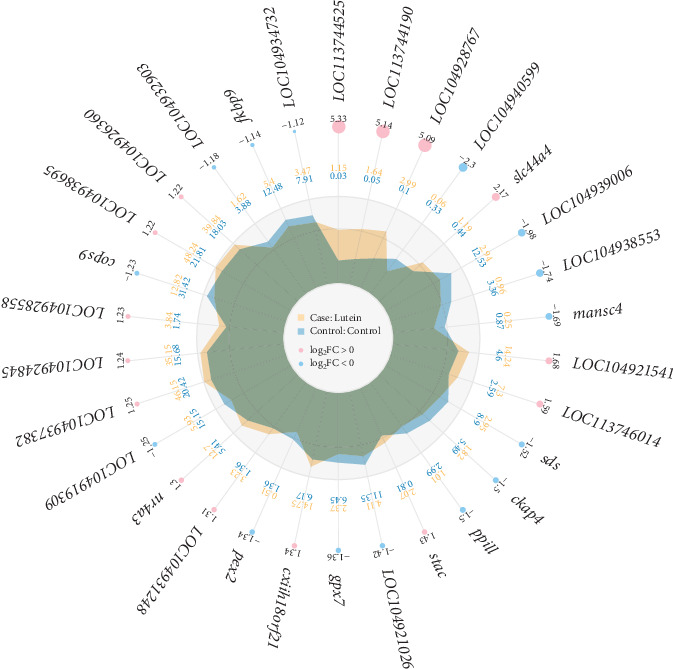
The differential expression radar image in different samples. The peripheral ring (light red) highlights upregulated genes, whereas downregulated genes are represented in light blue, with node sizes proportional to log_2_FC. The intermediate ring illustrates the experimental group's mean expression levels (outer band) versus the control group's (inner band). The central ring provides gene-specific expression values for both groups.

**Figure 5:**
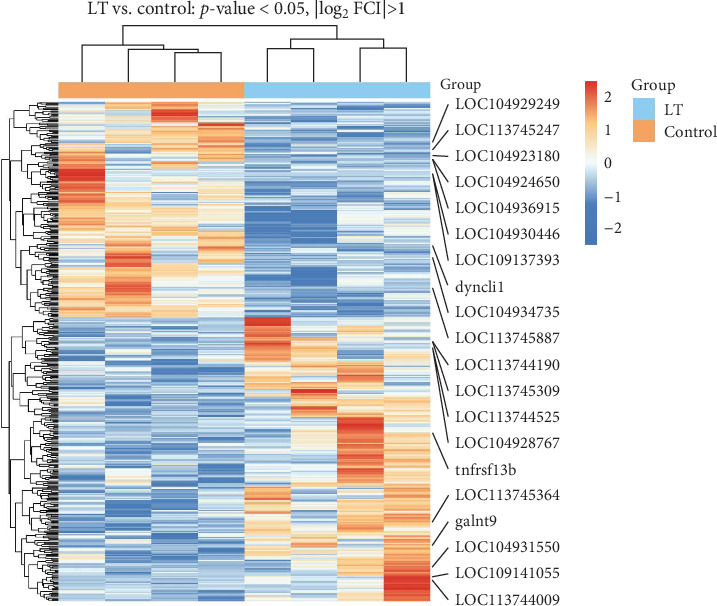
Heatmap of different gene expression in different samples. Red signifies protein-coding genes with relatively high expression levels, whereas blue denotes those exhibiting relatively low expression. The *x*-axis shows gene entries and the *y*-axis represents experimental samples. Genes are displayed along the *x*-axis, while experimental samples are arranged on the *y*-axis. Hierarchical clustering dendrogram branches partitioned the eight samples into distinct control and experimental groups, revealing systematic transcriptional divergence between cohorts. DEGs were screened under thresholds of |log_2_FC| > 1 and *p* < 0.05.

A total of 410 DEGs were identified between the control group and the LT group. Compared to the control group, 233 genes were upregulated and 177 genes were downregulated in the LT group. The radar plot of differential expression is shown in Figure 4, and the clustering heat map is presented in Figure 5. Notably, genes such as LOC104937478 (L-amino-acid oxidase, enables L-amino-acid oxidase activity), LOC104937491 (L-amino-acid oxidase-like, enables L-amino-acid oxidase activity), LOC104931550 (amine sulfotransferase, enables sulfotransferase activity), LOC113744190 (rab-3A-interacting protein-like, enables guanyl-nucleotide exchange factor activity), LOC104928767 (adhesion G protein-coupled receptor G2a, enables guanyl-nucleotide exchange factor activity), *tnfrsf13b*, and *galnt9*, among others, were significantly upregulated. In contrast, genes such as *tyrp1b*, *ptprnb*, *dync1i1*, LOC109137393 (Group 3 secretory phospholipase A2, enables phospholipase A2 activity), LOC104924650 (CD109 antigen, enables endopeptidase inhibitor activity), LOC113745247 (chromatin complexes subunit BAP18-like, promotes triple-negative breast cancer progression), and other were significantly downregulated.

#### 3.3.1. GO Annotation of DEGs

**Figure 6:**
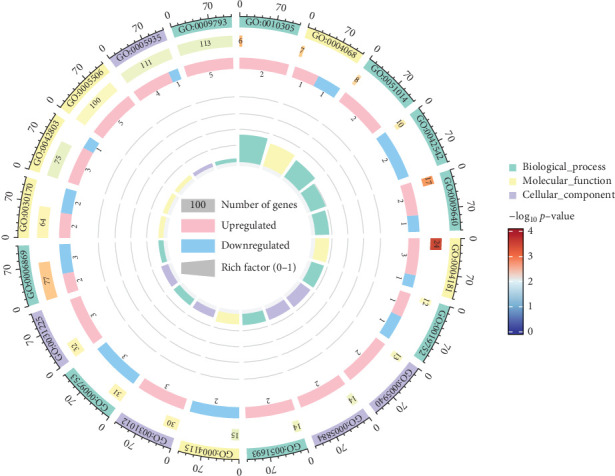
GO enrichment analysis circle of the 20 classifications in different samples. The first circle represents the classification of enrichment, with the outer circle indicating the gene count scale. Different colors correspond to distinct classifications. The second circle displays the number of genes in each classification and the associated *p*-value. Longer bars represent a greater number of genes, with the color intensity shifting to red for smaller *p*-values and blue for larger ones, with specific values shown below. The fourth circle depicts the RICHFACTOR values for each classification (the ratio of foreground genes to background genes), with background auxiliary lines at intervals of 0.2 for each bar.

Following differential gene analysis, DEGs were annotated using the GO database. DEGs between groups were performed according to genomic annotation information and comprehensive functional characterization. In the LT group, compared to the untreated controls, the upregulated GO terms in biological processes included leaf vascular tissue pattern formation and actin filament severing, while downregulated terms included response to jasmonic acid and response to hydrogen peroxide. In molecular function, upregulated GO terms included metallocarboxypeptidase activity and iron ion binding, while downregulated terms included inhibitor activity, 3′, 5′-cyclic-AMP phosphodiesterase (PDE) activity, and serine-type endopeptidase activity. In cellular components, upregulated GO terms included extracellular matrix and anchored component of the membrane, while downregulated terms included intracellular and extracellular regions.  Figure 6 presents the GO enrichment analysis circle diagram, highlighting the top 20 classifications with the smallest *p*-values.

#### 3.3.2. KEGG Pathway Analysis of Different Gene Expressions

**Figure 7:**
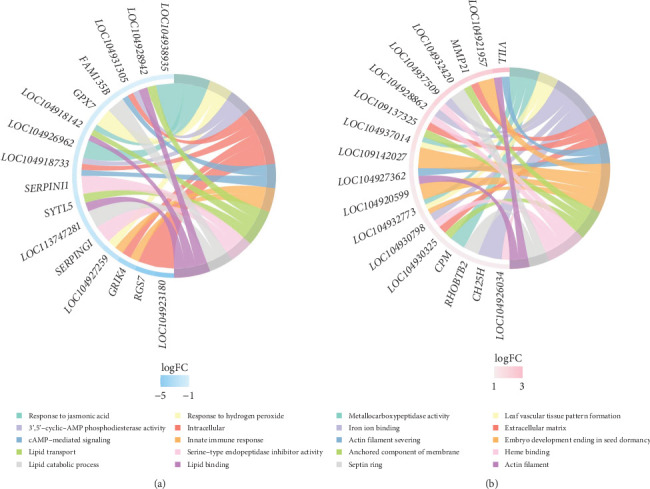
Enrichment analysis of related differential expression pathways and key differential genes. The enrichment analysis chord plot shows the top 10 categories with the smallest *p*-value. Subpart (A) shows the top 10 genes with the largest log_2_FC value in each category, and (B) the lateral heatmap represents the log_2_FC value of the corresponding gene.

A total of 122 KEGG pathways were significantly overrepresented in the DEGs between the LT and control groups. Of these, 66 pathways were upregulated, with eight pathways significantly upregulated, including phenylalanine, tyrosine, and tryptophan metabolic production, tyrosine metabolic pathway, and phenylalanine metabolic pathway. In contrast, 66 pathways were downregulated, with nine significantly downregulated, including melanogenesis and valine, leucine, and isoleucine biosynthesis. Additionally, 10 pathways exhibited partial upregulation and partial downregulation. The enrichment analysis of related differential expression pathways and key DEGs is presented in Figure 7.

### 3.4. Analysis of Differential Expressed Metabolites

**Figure 8:**
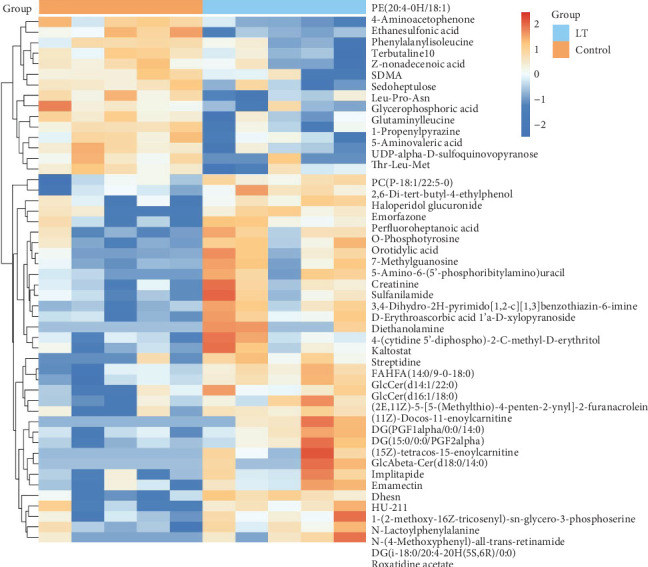
Heatmap of differentially expressed metabolites in different samples. Red indicates upregulation of metabolites and blue indicates downregulation of metabolites. Rows represent metabolites, while columns correspond to the different samples. Each row represents a significantly different metabolite from the top 50 with the smallest *p*-value.

In this study, a total of 3596 biochemical compounds were identified in the comparative analysis between experimental conditions and untreated controls, including 323 first-order metabolites, 1134 second-order metabolites, 165 third-order metabolites, and 1974 fourth-order metabolites. Compared to the control group, 99 metabolites, such as threonylalanine, citric acid, cetyl alcohol, and hypoxanthine, were significantly upregulated in the LT group, while 55 metabolites, including L-tyrosine, serine-glyoxylate, 4-aminoacetophenone, and isonicotinic acid, were significantly downregulated. The cluster heat map of the top 50 significant DEMs with the smallest *p*-values is shown in Figure 8.

#### 3.4.1. KEGG Pathway Analysis of Differentially Expressed Metabolites

**Figure 9:**
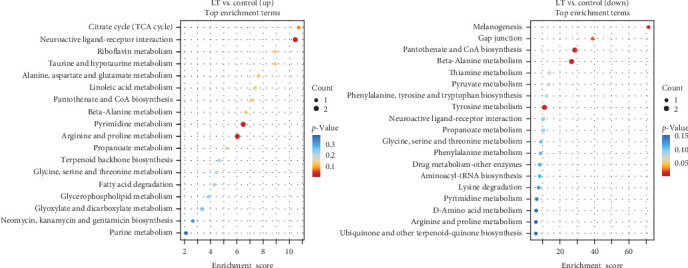
KEGG enrichment analysis of significant pathways in different samples. The vertical axis is the top 20 pathways and the horizontal axis is the enrichment score. The size of the bubble represents the number of differential metabolites contained, and the redder the color, the smaller the *p*-value, and the more significant the difference. The upregulated pathways mainly include TCA cycle, pyrimidine metabolism, purine metabolism, and tricarboxylic acid cycle metabolism. The downregulated pathways include melanogenesis, phenylalanine, tyrosine and tryptophan biosynthesis and tyrosine metabolism.

KEGG pathway enrichment analysis of the significant DEMs identified 18 upregulated pathways and 19 downregulated pathways. Upregulated pathways included signaling molecules and interaction, nucleotide metabolic pathway, amino acid metabolic pathway, and metabolic pathway of other amino acids. Downregulated pathways included pantothenate and CoA biosynthesis, beta-alanine metabolic pathway, tyrosine metabolic pathway, melanogenesis, gap junction, and thiamin metabolism, among others. The 20 pathways with the minimal *p*-values are listed in Figure 9.

### 3.5. Combined Transcriptomics/Metabolomics Analysis

**Figure 10:**
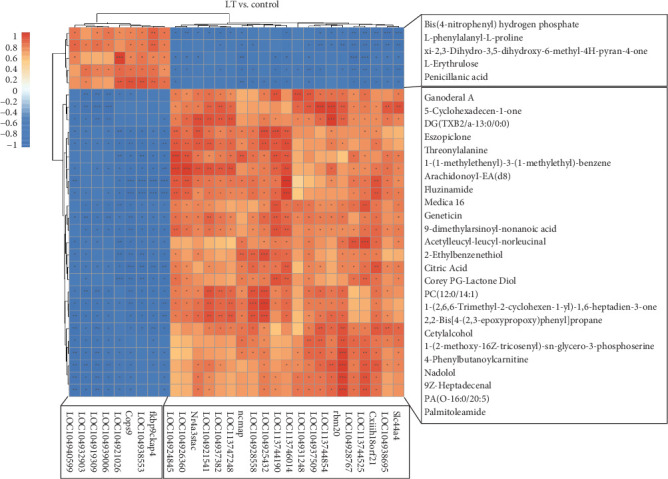
The heatmap for correlation analysis of different gene expression and differentially expressed metabolites. The vertical axis is the differential metabolites and the horizontal axis is the differentially expressed genes. Red is difference upregulated, blue is difference downregulated. The top 30 entries with significant differences between the transcriptome and the metabolome were calculated, with “*⁣*^*∗*^” indicating correlation *p* < 0.005, “*⁣*^*∗∗*^” indicating correlation *p* < 0.01, and “*⁣*^*∗∗∗*^” indicating correlation *p* < 0.001.

Correlation analysis between DEGs and DEMs from transcriptomics and metabolomics identified the top 30 most significantly altered items, with their correlation coefficients calculated and visualized in a heatmap (Figure 10). DEGs and DEMs were mapped to the KEGG pathways, revealing their collective involvement in 14 pathways, including linoleic acid metabolism, alanine, tyrosine, tryptophan biosynthesis, tyrosine metabolism, melanogenesis, and phenylalanine metabolism, as listed in Table 6.

### 3.6. qRT-PCR Analysis

**Figure 11:**
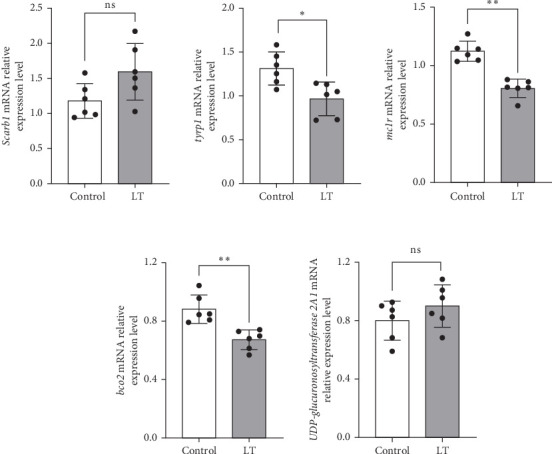
Relative expression of differentially expressed genes in the ventral skin of *Larimichthys crocea*. “*⁣*^*∗*^” indicates significant differences (*p* < 0.05), while “*⁣*^*∗∗*^” represents highly significant differences (*p* < 0.01) and “ns” indicates no significant differences (*p＞*0.05). Bar graphs represent the means and standard error means of six biological replicates, with each replicate subjected to three technical measurements. *scarb1*: *p* = 0.0569, *tyrp1b*: *p* = 0.0101, *mc1r*: *p＜*0.0010, *bco2*: *p* = 0.0015, *UDP-glucuronosyltransferase 2A1*: *p* = 0.2431.

Genes associated with skin pigment synthesis, including *scarb1*, *tyrp1b*, *mc1r*, *bco2*, and *UDP-glucuronosyltransferase 2A1*, were selected for qRT-PCR validation based on high-throughput sequencing results. The qRT-PCR data experimentally validated the accuracy of the sequencing results. Gene expression levels are presented in Figure 11.

### 3.7. Analysis of Specific Content of Lutein and Astaxanthin in *Larimichthys crocea* Skin

**Figure 12:**
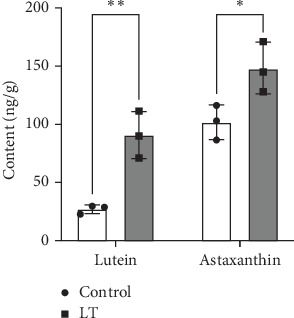
The content of lutein and astaxanthin in different samples. “*⁣*^*∗*^” indicates significant differences (*p* < 0.05), while “*⁣*^*∗∗*^” represents highly significant differences (*p* < 0.01). Bar graphs represent the means and standard error means of three biological replicates, with each replicate subjected to three technical measurements.

The contents of lutein and astaxanthin in the ventral skin of *L. crocea* from both the control and experimental groups were measured using LC-MS. As shown in Table 7 and Figure 12, the LT group exhibited significantly higher levels of lutein and astaxanthin relative to the control group.

## 4. Discussion

*Larimichthys crocea* is highly prized by consumers for its tender flesh and rich nutritional value, making it a popular species in recent cage aquaculture practices. The color of this fish is a crucial determinant of its economic value [27]. However, the variation in breeding environments often leads to a decline in body color in farmed *L. crocea*, significantly reducing its market value. Studies have demonstrated that supplementing feeds with appropriate additives can enhance the body color of farmed animals [28–31]. Marigold, a traditional Chinese herbal medicine, is rich in lutein, which has been shown to not only improve body color but also enhance the immune function of farmed animals [32–35].

Dietary supplementation with three pigment additives can not significantly alter growth parameters in *L. crocea* over the 28-day trial. FBW, PWG, and SGR showed no statistically significant differences (*p* > 0.05). Short-term administration of pigment additives exerted no adverse effects on feeding behavior in *L. crocea*.

### 4.1. Effect of *Tagetes erecta* on Color Metrics in *Larimichthys crocea*

The *L*^⁣^*∗*^^, *a*^*∗*^, and *b*^*∗*^ values are commonly employed to quantify fish coloration [36–38]. After 28 days of feeding *L. crocea* with three kinds of pigment supplements, ventral skin *L*^⁣^*∗*^^, *a*^*∗*^, and *b*^*∗*^ values were measured using a spectrophotometer, and CV and HV were subsequently calculated. In comparison to the control and other treatment groups, the LT group exhibited significantly higher chroma and a more yellowish hue under various conditions. Based on these findings, the LT group was selected for further molecular-level investigations.

### 4.2. *Tagetes erecta* Regulates the Carotenoid Uptake and Metabolism Genes

Carotenoids, which share spectral similarities with pteridines, are responsible for producing red to yellow pigmentation in animals. These compounds are abundantly found in the red or yellow tissues of fish [39, 40]. In *L. crocea*, the yellow coloration of the body surface largely depends on carotenoid deposition.

The transport of carotenoids in the bloodstream is primarily facilitated by lipoproteins [41]. Among the key genes involved in carotenoid absorption and transport, *ABCA1* plays a significant role, while *SCARB1* is directly correlated to carotenoid pigmentation in vertebrate species, mediating their absorption and transport [42, 43]. Existing studies have indicated that both *ABCA1* and *SCARB1* are highly expressed in all-red Oujiang carp compared to their all-white counterparts [44]. The *ABCA12* gene plays a crucial role in the translocation of lipid molecules, including ceramide, and plays a role in forming an extracellular lipid layer in the stratum corneum, contributing to skin barrier function [45]. In the present study, high-throughput sequencing revealed a significant upregulation of *ABCA12* in the LT group (fold change = 2.7772, *p* = 0.0425) compared to the control group, with no significant difference observed in *SCARB1* expression (fold change = 1.2501). However, qRT-PCR validation confirmed a slight upregulation of *SCARB1* in the LT group (*p* = 0.0569), though not statistically significant. These results suggest that the more pronounced yellow coloration observed in the LT group may result from the upregulation of *ABCA12* and *SCARB1*, which could enhance the transport, uptake, and deposition of carotenoids in the fish's skin.

Carotenoid oxygenases are crucial in carotenoid degradation, with three primary types: carotenoid isomerooxygenase (*NinaB*), β,β-carotene 15,15'-monooxygenase (*BCMO*), and β,β-carotene 9',10'-oxygenase (*BCO2*) [46]. *BCO2* catalyzes the asymmetric cleavage of the 9'-10' double bond in the polyene backbone of carotenoids. It encodes a carotenoid lyase that mediates the cleavage of dietary carotenoids, preventing excessive carotenoid accumulation in the body, metabolizing lutein, and serving as an alternative pathway for retinoic acid synthesis [47–49]. In this study, qRT-PCR exhibited statistically significant decreases in *BCO2* expression in the ventral skin of experimental fish in the LT group (fold change = 0.4798, *p* = 0.0015), suggesting a blockade in carotene degradation and subsequent deposition in the ventral skin.

Cytochrome P450 (*CYP450*) enzymes are extensively expressed across species and play key roles in pigment biosynthesis. For instance, cytochrome P450 Cit CYP97B regulates carotenoid diversity in citrus by hydroxylating β-cryptoxanthin [50]. Mundy et al. [51] demonstrated that the *CYP 2J2*-like gene cluster is essential for the coloration of red ketone carotenoids, while Xu et al. [52] proposed that *CYP 2J2* promotes carotenoid deposition, particularly yellow pigments, via the arachidonic acid metabolic105and lipid pathways. *CYP 26A1* is a retinoic acid-degrading enzyme [53–56], enhances melanin production [57]. In this study, high-throughput sequencing revealed significant upregulation of *CYP 2J2* (fold change = 2.0239, *p* = 0.0384) and *CYP 26A1* (fold change = 3.9715, *p* = 0.0119) in the LT group. These findings suggest that the enhanced yellow pigmentation in the LT group may result from increased carotenoid deposition due to upregulation of *CYP 2J2*, while the concurrent elevation of *CYP 26A1* likely accelerates retinoic acid degradation, reducing melanin deposition.

### 4.3. *Tagetes erecta* Regulates the Melanogenesis and Metabolism Related Genes

Carotene coloration is influenced by melanocyte distribution [58]. Tyrosinase, a rate-limiting enzyme in controlling melanogenic, serves as a critical modulator of fish skin color. Previous studies have demonstrated a correlation between the activity of tyrosinase and the content of melanin in fish, with factors such as genetics, nutrition, differences in the environment, and developmental stages impacting this regulation [59–61]. Melanin production can also be inhibited by certain compounds that target the *RAS/RAF/MAPK* cascade or the *cAMP*–*PKA*–cAMP response element binding protein (*CREB*) signaling pathway [62–64]. cAMP modulates the activity of tyrosinase, the eumelanin-specific enzyme tyrosinase-related protein 1 (*Tyrp1*), and dopachrome tautomerase (*Dct*). Under normal conditions, these enzymes facilitate the deposition of eumelanin (brown to black pigment) in melanosomes. A decrease in intracellular cAMP levels shifts pigment production toward pheomelanin (yellow to red pigment) [65]. *MC1R*, expressed in melanocytes, activates intracellular cAMP levels, which in turn stimulates melanogenesis via *α-MSH* and the transcription of tyr, leading to increased melanin synthesis [66, 67]. PDE play a key role in regulating cAMP levels [68]. The level of intracellular cAMP is regulated by the antagonism of *PDE*, which can reduce cAMP into 5'-AMP [69, 70]. Transcription factors such as *CREB* and cAMP response element modulator (*CREM*) modulate gene transcription in response to increased cAMP levels. In this study, high-throughput sequencing revealed a significant reduction in *MC1R* expression (fold change = 0.4113, *p* = 0.0273), downregulation of pro-opiomelanocortin, which directs *a-MSH* synthesis (fold change = 0.4623, *p* = 0.0009), and a decrease in cAMP-specific 3',5'-cyclic PDE 4A (*PDE4A*) expression (fold change = 0.3300, *p* = 0.0060). The expression of high-affinity cGMP-specific *PDE9A* was significantly reduced (fold change = 0.4625, *p* = 0.0187). This reduction may be attributed to decreased MC1R expression, which impairs the normal activation of the cAMP signaling pathway, leading to reduced α-MSH synthesis. Consequently, the diminished demand for cAMP degradation results in downregulation of both *PDE4A* and *PDE9A*. Transcriptomic and qRT-PCR analyses also revealed a significant downregulation of *tyrp1b* (fold change = 0.3453, *p* = 0.0128). This suggests that the cAMP pathway's downregulation fails to activate the key melanin synthesis gene, *tyrp1b*, leading to reduced eumelanin expression on the body surface, increased pheomelanin production, and a visible alteration in the body color of *L. crocea*. Additionally, the significant upregulation of cAMP response element modulator a (*crema*; fold change = 2.3704. *p＜*0.0010) may help compensate for the reduced cAMP levels, thus, balancing transcriptional responses.

### 4.4. Effect of *Tagetes erecta* in *Larimichthys crocea*: Dual Regulation of Citrate and Tyrosine Production

Citric acid, an intermediate in the tricarboxylic acid (TCA) cycle, plays a pivotal role in energy metabolism, macromolecular synthesis, and maintaining cellular redox balance [71]. Tributyl citrate has demonstrated antimelanin activity [72], and citric acid has been identified as a depigmenting agent in topical treatments for hyperpigmentation [73]. To validate the melanogenesis-inhibitory properties of succinic and citric acids, multiple studies have used B16-F10 cells to evaluate cell viability and melanin content. These studies demonstrated that treatment with succinic and citric acids resulted in a reduction of melanogenic activity [74].

In the nontargeted metabolomic analysis of this study, citric acid was significantly upregulated as a DEM (fold change = 1.7589), which may be attributed to the addition of lutein in the feed. This increase in citric acid synthesis in *L. crocea* could inhibit melanin production. L-tyrosine, a precursor of catecholamines and melanin, is hydroxylated by tyrosinase to L-dihydroxyphenylalanine (L-DOPA), which is further oxidized to dopaquinone and then to melanin [75]. Tyrosine levels may influence the transition of melanocytes from a proliferative to a melanin-synthesizing and terminally differentiated state [76]. In this study, L-tyrosine was significantly downregulated (fold change = 0.6793), which likely blocked melanin synthesis in the skin of *L. crocea*, enhancing the prominence of yellow pigments on the skin.

### 4.5. Transcriptome-Metabolome Analysis: *Tagetes erecta* Improve Skin Pigmentation in *Larimichthys crocea* Through Tyrosine and Melanin Production Pathways

**Figure 13:**
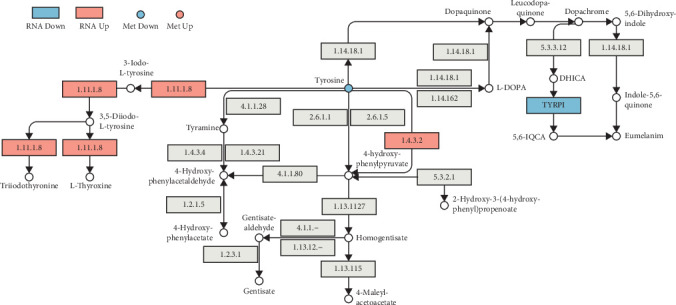
Tyrosine-related metabolism in the skin of *Larimichthys crocea* after lutein treatment. Blue indicates downregulated, red indicates upregulated, and gray indicates genes detected but not statistically significant; boxes are DEGs and circles are DEMs. The details of the difference items in the figure are: [1.11.1.8]: LOC104928854, log_2_FC: 2.80; [1.4.3.2]: LOC104937491, log_2_FC: 2.74, Loc104937478, log_2_FC: 2.61; [*TYRP1*]: LOC104930927, log_2_FC: −1.53; Tyramine: *L-Tyrosine*; log_2_FC: −0.50.

Mapping DEGs identified by high-throughput sequencing and metabolites detected by nontargeted metabolomics to KEGG pathways revealed that lutein upregulated key enzymes involved in tyrosine-related metabolism (Figure 13), such as L-amino acid oxidase [EC: 1.4.3.2] (genes LOC1049374780 and LOC104937491). This upregulation accelerates the conversion of tyrosine away from eumelanin synthesis. Simultaneously, downregulation of *TYRP1* expression inhibited eumelanin production, thus, reinforcing the yellow pigmentation characteristic on the surface of *L. crocea*.

**Figure 14:**
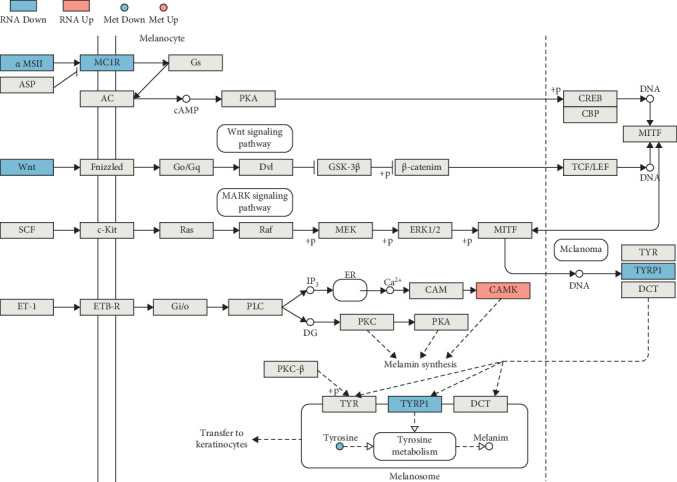
Differentially expressed genes and differentially expressed metabolites mapped to the MELANOGENESIS pathway through joint analysis. Blue indicates downregulated, red indicates upregulated, and gray indicates genes detected but not statistically significant; boxes are DEGs and circles are DEMs. The details of the difference items in the figure are: [*α-MSH*]: LOC104921984, log_2_FC: −1.11; [*MC1R*]: *mc1r*, log_2_FC: −1.28; [*Wnt*]: wnt10a, log_2_FC: −1.93; [*TYRP1*]: LOC104930927, log_2_FC: −1.53; [*CAMK*]: *camk2b*, log_2_FC: 1.48; [*Tyrosine*]: *L-Tyrosine*, log_2_FC: −0.50.

In the melanin production pathway (Figure 14), significant downregulation of *MC1R*, pro-opiomelanocortin (LOC104921984), which guides the synthesis of*α-MSH*, as well as *Wnt10a*, *Tyrp1*, and tyrosine-key regulators of melanin synthesis-resulted in a reduction of the entire melanin biosynthesis pathway (*p* = 0.0231). This led to decreased melanin synthesis and deposition, thereby accentuating the yellow pigment on the surface of *L. crocea*.

## 5. Conclusions

This study concludes that *T. erecta* supplementation significantly enhances the body color of *L. crocea*, compared with the astaxanthin feeding group and the amaranth feeding group, the *T. erecta* feeding group had the best effect on enhancing the yellow skin of large yellow croaker. The potential mechanism may involve the upregulation of carotenoid deposition and the downregulation of the expression of tyrosine, an important precursor in the melanin formation pathway. Therefore, the contribution of carotenoids to coloring became more prominent, thereby improving the body color of *L. crocea*. Critically, our findings demonstrate that dietary pigment additives, particularly lutein from *T. erecta*, enhance golden skin coloration in *L. crocea*, directly increasing market value.

## Figures and Tables

**Table 1:** Primer sequences used for qRT-PCR analysis.

Gene name	Primer sequence	Product length
*scarb1*	GGACTACGCCCCCAACGA	100
	AGGATGAGAGATGAAGCACGGA	

*TYRP1*	ATTGAAGGCTACAGCGCCCC	90
	GCCCGTCCCGTTGAGGAAAA	

*mc1r*	ACCTCACCACGGGAGAACGA	149
	CATGGGCGAATGCAGGTTGC	

*Bco2*	TGCCCTCCCGATGTCAACCT	250
	GTCGGGCCGGTTGTTCAAGT	

*UDP-glucuronosyltransferase 2A1*	AAGTTTGAACTCACACATGCAAAGA	77
	GCTCAGAGTGTCAACAGTAATCCT	

*β-Actin*	ATTGCCCCACCTGAGCGTAAA	183
	GTTGGGTGTTTGGTTGGGGAGG	

**Table 2:** Gradient program of mobile phase.

Time (min)	Ratio (%)	
	Mobile phase A	Mobile phase B
0	95	5
2	95	5
4	70	30
8	50	50
10	20	80
14	0	100
15	0	100
15.1	95	5
16	95	5

**Table 3:** MS/MS parameters for lutein and astaxanthin.

Compound name	Q1 (m/z)	Q3 (m/z)	CE (V)
Lutein	551.3	119.1/145.1/175.3	10/43/25
Astaxanthin	597.3	579.1/147	17/26

**Table 4:** Growth performance of *Larimichthys crocea* fed dietary additives.

Parameters	Control	LT	XT	AT
FBW (g)	70.99 ± 7.25	69.64 ± 4.49	68.85 ± 6.63	69.90 ± 7.87
PWG (%)	117.30 ± 22.20	113.17 ± 13.74	110.74 ± 20.30	113.96 ± 24.10
SGR (%/d)	2.74 ± 0.37	2.68 ± 0.23	2.63 ± 0.34	2.68 ± 0.40

*Note:* Values are presented as mean ± standard deviation. Number of replicates is 12.

Abbreviations: FBW, final body weight; PWG, percent weight gain, SGR, specific growth ratio.

**Table 5:** Effects of dietary pigment additives on colorimetric parameters of ventral skin.

Treatment	Parameters	Group	Measurement position		
			A1	A2	A3
Dark	CV	Control	52.13 ± 3.82	32.44 ± 5.02^a^	53.12 ± 9.45^a^
		LT	59.74 ± 5.48	43.10 ± 9.46^ab^	36.45 ± 7.18^b^
		XT	59.52 ± 8.73	35.51 ± 4.33^a^	43.45 ± 3.43^ab^
		AT	58.21 ± 9.67	56.72 ± 17.07^b^	39.71 ± 4.99^b^
	HV	Control	1.47 ± 0.02^a^	1.54 ± 0.01^a^	1.47 ± 0.01^a^
		LT	1.43 ± 0.02^ab^	1.49 ± 0.03^b^	1.50 ± 0.02^b^
		XT	1.43 ± 0.02^ab^	1.51 ± 0.01^ab^	1.50 ± 0.01^b^
		AT	1.41 ± 0.01^b^	1.44 ± 0.03^c^	1.48 ± 0.01^ab^

Light	CV	Control	44.24 ± 11.35^a^	32.74 ± 9.42^a^	22.68 ± 7.88
		LT	61.77 ± 13.48^b^	56.84 ± 5.93^b^	33.29 ± 11.26
		XT	35.59 ± 5.61^a^	28.39 ± 10.27^a^	24.47 ± 11.15
		AT	45.12 ± 8.01^a^	39.88 ± 3.77^a^	26.88 ± 6.32
	HV	Control	1.42 ± 0.03	1.43 ± 0.01^a^	1.50 ± 0.02^a^
		LT	1.43 ± 0.00	1.47 ± 0.01^b^	1.54 ± 0.01^b^
		XT	1.44 ± 0.02	1.44 ± 0.01^a^	1.50 ± 0.02^a^
		AT	1.45 ± 0.03	1.47 ± 0.01^b^	1.52 ± 0.01^ab^

*Note:* Values are presented as mean ± standard deviation. Different superscript letters within each data arrangement represent statistically significant variations between treatment conditions (*p* < 0.05). Number of replicates is 4. CV represents the perpendicular distance from the measured color point to the lightness axis, with higher CV values indicating greater color saturation; HV denotes color categories (red, orange, green, blue, and purple), where 0° corresponds to red, 90° to yellow, 180° to green, and 270° to blue.

Abbreviations: CV, chroma value; HV, hue value.

**Table 6:** Tyrosine and tryptophan biosynthesis, tyrosine metabolism, melanogenesis, and other 14 pathways.

ID	Term	Classification level 1	Classification level 2
ko00591	Linoleic acid metabolism	Metabolism	Lipid metabolism
ko00250	Alanine, aspartate, and glutamate metabolism	Metabolism	Amino acid metabolism
ko00400	Phenylalanine, tyrosine, and tryptophan biosynthesis	Metabolism	Amino acid metabolism
ko00350	Tyrosine metabolism	Metabolism	Amino acid metabolism
ko04916	Melanogenesis	Organismal systems	Endocrine system
ko00360	Phenylalanine metabolism	Metabolism	Amino acid metabolism
ko00410	Beta-alanine metabolism	Metabolism	Metabolism of other amino acids
ko04080	Neuroactive ligand–receptor interaction	Environmental information processing	Signaling molecules and interaction
ko00770	Pantothenate and CoA biosynthesis	Metabolism	Metabolism of cofactors and vitamins
ko00260	Glycine, serine, and threonine metabolism	Metabolism	Amino acid metabolism
ko00983	Drug metabolism-other enzymes	Metabolism	Xenobiotics biodegradation and metabolism
ko00564	Glycerophospholipid metabolism	Metabolism	Lipid metabolism
ko00071	Fatty acid degradation	Metabolism	Lipid metabolism
ko00310	Lysine degradation	Metabolism	Amino acid metabolism

**Table 7:** The content of lutein and astaxanthin in different samples.

Item	Weight (mg)	Sample ID	Area	Content, (ng/g)	Ave.	Error bar
Lutein	204.90	C1	2585.00	22.89	27.07	2.10
	204.90	C2	3346.00	29.58		
	202.40	C3	3211.00	28.74		
	204.00	L1	8013.00	70.90	90.81	11.72
	201.50	L2	10060.00	90.07		
	196.90	L3	12170.00	111.46		

Astaxanthin	204.90	C1	4660.00	116.09	101.79	8.59
	204.90	C2	2810.00	86.38		
	202.40	C3	3760.00	102.89		
	204.00	L1	6410.00	144.83	147.99	12.64
	201.50	L2	5260.00	127.85		
	196.90	L3	7680.00	171.28		

## Data Availability

The data that support the findings of this study are available from the corresponding author upon reasonable request.

## References

[1] Suo N., Wu Y., Zhou Z. (2022). Genome-Wide Association and Expression Analysis Revealed the Candidate Variants and Molecular Underpinnings of Cold-Stress Response in Large Yellow Croaker. *Marine Biotechnology*.

[2] Mao J., Fu J., Qi X., Chen Y., Zhang B. (2023). Effect of Theaflavins on the Quality of Large Yellow Croaker, *Larimichthys Crocea*, During Refrigerated Storage. *Journal of the Science of Food and Agriculture*.

[3] Sun P., Bao P., Tang B. (2017). Transcriptome Analysis and Discovery of Genes Involved in Immune Pathways in Large Yellow Croaker (*Larimichthys Crocea*) Under High Stocking Density Stress. *Fish and Shellfish Immunology*.

[4] Ma R., Meng Y., Zhang W., Mai K. (2020). Comparative Study on the Organoleptic Quality of Wild and Farmed Large Yellow Croaker *Larimichthys Crocea*. *Journal of Oceanology and Limnology*.

[5] Yi X., Shen H., Li J. (2018). Effects of Dietary Vitamin E and Astaxanthin on Growth, Skin Colour and Antioxidative Capacity of Large Yellow Croaker *Larimichthys Crocea*. *Aquaculture Nutrition*.

[6] Zheng J. L., Wan F. G., Chen Y. L. (2024). Comparative Study on the Morphological Characteristics, Nutritional Quality, and Tastes of Large Yellow Croaker From Five Cage Culture Areas: Relay Farming Improved Fish Quality. *Aquaculture*.

[7] Nord H., Dennhag N., Muck J., Hofsten J. V. (2016). Pax7 Is Required for Establishment of the Xanthophore Lineage in Zebrafish Embryos. *Molecular Biology of the Cell*.

[8] Torrissen O., Hardy R., Shearer K., Scott T., Stone F. E. (1990). Effect of Dietary Canthaxanthin Level and Lipid Level on Apparent Digestibility Coefficients for Canthaxanthin in Rainbow Trout (*Oncorhynchus Mykiss*). *Aquaculture*.

[9] Kalinowski C. T., Robaina L. E., Fernández-Palacios H., Schuchardt D., Izquierdo M. S. (2005). Effect of Different Carotenoid Sources and Their Dietary Levels on Red Porgy, *Pagrus Pagrus*, Growth and Skin Colour. *Aquaculture*.

[10] Yi X., Xu W., Zhou H. (2014). Effects of Dietary Astaxanthin and Xanthophylls on the Growth and Skin Pigmentation of Large Yellow Croaker *Larimichthys Croceus*. *Aquaculture*.

[11] Yi X., Li J., Xu W. (2015). Shrimp Shell Meal in Diets for Large Yellow Croaker *Larimichthys Croceus*: Effects on Growth, Body Composition, Skin Coloration and Anti-Oxidative Capacity. *Aquaculture*.

[12] Langi P., Kiokias S., Varzakas T., Proestos C. (2018). Carotenoids: From Plants to Food and Feed Industries: Methods and Protocols.

[13] Sanjaya S. S., Park M. H., Karunarathne W. A. H. M. (2024). Inhibition of A-Melanocyte-Stimulating Hormone-Induced Melanogenesis and Molecular Mechanisms by Polyphenol-Enriched Fraction of *Tagetes Erecta* L. Flower. *Phytomedicine*.

[14] Sarker U., Oba S. (2019). Protein, Dietary Fiber, Minerals, Antioxidant Pigments and Phytochemicals, and Antioxidant Activity in Selected Red Morph, *Amaranthus*, Leafy Vegetable. *PLOS ONE*.

[15] Baraniak J., Kania-Dobrowolska M. (2022). The Dual Nature of Amaranth—Functional Food and Potential Medicine. *Foods*.

[16] Swian H. S., Senapati S. R., Meshram S. J., Mishra R., Murthy S. J. (2014). Effect of Dietary Supplementation of Marigold Oleoresin on Growth, Survival and Total Muscle Carotenoid of Koi Carp, *Cyprinus Carpio* L.. *Journal of Applied and Natural Science*.

[17] Biswas P., Singh S. K., Debbarma R. (2024). Effects of Carotenoid Supplementation on Colour, Growth and Physiological Function of the Endemic Dwarf Chameleon Fish (*Badis Badis*). *Journal of Animal Physiology and Animal Nutrition*.

[18] Pailan G. H., Sardar P., Mahapatra B. K. (2015). Marigold Petal Meal: A Natural Carotenoid Source for Pigmentation in Swordtail (Xiphophorus Helleri). *Animal Nutrition and Feed Technology*.

[19] Zhang Z., Shi C., Han J. (2024). Nonvisual System-Mediated Body Color Change in Fish Reveals Nonvisual Function of Opsin 3 in Skin. *Journal of Photochemistry and Photobiology B: Biology*.

[20] Liu S. J., Song S. H., Wang W. Q., Song S. Q. (2015). De Novo Assembly and Characterization of Germinating Lettuce Seed Transcriptome Using Illumina Paired-End Sequencing. *Plant Physiology and Biochemistry*.

[21] Chen S., Zhou Y., Chen Y., Jia G. (2018). Fastp: An Ultra-Fast All-in-One Fastq Preprocessor. *Bioinformatics*.

[22] Kim D., Langmead B., Salzberg S. L. (2015). HISAT: A Fast Spliced Aligner With Low Memory Requirements. *Nature Methods*.

[23] Roberts A., Trapnell C., Donaghey J., Rinn J. L., Pachter L. (2011). Improving RNA-Seq Expression Estimates by Correcting for Fragment Bias. *Genome Biology*.

[24] Simon A., Theodor P. P., Wolfgang H. (2015). HTSeq-a Python Framework to Work With High-Throughput Sequencing Data. *Bioinformatics*.

[25] Carbon S., Douglass E., Dunn N., Good B., Harris N. L., Lewis S. E. (2019). The Gene Ontology Resource: 20 Years and Still Going Strong. *Nucleic Acids Research*.

[26] Minoru K., Michihiro A., Susumu G. (2007). Kegg for Linking Genomes to Life and the Environment. *Nucleic Acids Research*.

[27] Vissio P. G., Darias M. J., Di Yorio M. D. P., Pérez Sirkin D. I., Delgadin T. H. (2021). Fish Skin Pigmentation in Aquaculture: The Influence of Rearing Conditions and its Neuroendocrine Regulation. *General and Comparative Endocrinology*.

[28] Zhang J., Tian C., Zhu K. (2023). Effects of Natural and Synthetic Astaxanthin on Growth, Body Color, and Transcriptome and Metabolome Profiles in the Leopard Coralgrouper (*Plectropomus Leopardus*). *Animals*.

[29] Saez M. I., Galafat A., Suarez M. D. (2023). Effects of Raw and Hydrolysed, *Nannochloropsis Gaditana*, Biomass Included at Low Level in Finishing Diets for Gilthead Seabream (*Sparus Aurata*) on Fillet Quality and Shelf Life. *Journal of Applied Phycology*.

[30] Li J. B., Wang Z., Cao X. F. (2023). Effects of Supplemental Mixed Bile Acids on Growth Performance, Body Composition, Digestive Enzyme Activities, Skin Color, and Flesh Quality of Juvenile Large Yellow Croaker *Larimichthys Crocea* in Soybean Oil Based Diet. *Frontiers in Marine Science*.

[31] Pezeshk F., Babaei S., Kenari A. A., Hedayati M., Naseri M. (2019). The Effect of Supplementing Diets With Extracts Derived From Three Different Species of Macroalgae on Growth, Thermal Stress Resistance, Antioxidant Enzyme Activities and Skin Colour of Electric Yellow Cichlid (*Labidochromis Caeruleus*). *Aquaculture Nutrition*.

[32] Yuangsoi B., Jintasataporn O., Areechon N., Tabthipwon P. (2011). The Pigmenting Effect of Different Carotenoids on Fancy Carp (*Cyprinus Carpio*). *Aquaculture Nutrition*.

[33] Besen K. P., Melim E. W. H., Cunha L. D., Favaretto E. D., Moreira M., Fabregat T. E. H. P. (2019). Lutein as a Natural Carotenoid Source: Effect on Growth, Survival and Skin Pigmentation of Goldfish Juveniles *Carassius Auratus*. *Aquaculture Research*.

[34] Shanmugasundaram R., Selvaraj R. K. (2011). Dietary Lutein and Fish Oil Interact to Alter Atherosclerotic Lesions in a Japanese Quail Model of Atherosclerosis. *Journal of Animal Physiology and Animal Nutrition*.

[35] Yousefi M., Adineh H., Ghafarifarsani H., Raeeszadeh M., Farsani M. N., Hashemianfar S. A. M. (2024). Immunological, Antioxidant, Growth Responses, and Disease Resistance of Rainbow Trout, *Oncorhynchus Mykiss*, With Feeding Diets Supplemented With *Lactobacillus Salivarius* and Lutein. *Annals of Animal Science*.

[36] McLean E. (2021). Fish Tank Color: An Overview. *Aquaculture*.

[37] Dijkstra P. D., Maguire S. M., Harris R. M. (2017). The Melanocortin System Regulates Body Pigmentation and Social Behaviour in a Colour Polymorphic Cichlid Fish. *Proceedings of the Royal Society B-Biological Sciences*.

[38] Border S., Piefke T., Fialkowski R. (2019). Color Change and Pigmentation in a Color Polymorphic Cichlid Fish. *Hydrobiologia*.

[39] Li H. X., Tyndale S. T., Heath D. D., Letcher R. J. (2005). Determination of Carotenoids and All-*Trans*-Retinol in Fish Eggs by Liquid Chromatography-Electrospray Ionization-Tandem Mass Spectrometry. *Journal of Chromatography B-Analytical Technologies in the Biomedical and Life Sciences*.

[40] Liu S. Y., Li Y. L., Wu Y. N., Bell L. N., Davis D., Wang Y. F. (2012). Extraction, Identification and Quantitation of Carotenoids in Discolored Channel Catfish (*Ictalurus Punctatus*)Fillets. *Journal of Food Composition and Analysis*.

[41] Reboul E. (2019). Mechanisms of Carotenoid Intestinal Absorption: Where Do We Stand?. *Nutrients*.

[42] Saunders L. M., Mishra A. K., Aman A. J., Lewis V. M., Parichy D. M. (2019). Thyroid Hormone Regulates Distinct Paths to Maturation in Pigment Cell Lineages. *eLife*.

[43] Kanika N. H., Ke J. P., Mandal R. N., Wang J., Wang C. (2023). Comparative Transcriptome and Metabolome Analyses of Wild and Mutant Oujiang Color Common Carp Through Editing, *Scarb1*, Gene by CRISPR/Cas Technology. *Aquaculture*.

[44] Du J., Chen H., Mandal B. K., Wang J., Wang C. (2021). HDL Receptor/Scavenger Receptor B1-Scarb1 and Scarb1-Like Mediate the Carotenoid-Based Red Coloration in Fish. *Aquaculture*.

[45] Akiyama M. (2014). The Roles of ABCA12 in Epidermal Lipid Barrier Formation and Keratinocyte Differentiation. *Biochimica et Biophysica Acta (BBA) - Molecular and Cell Biology of Lipids*.

[46] Sun Y. Y., Liu M. F., Yan C. C. (2020). CRISPR/Cas9-Mediated Deletion of B, B-Carotene 9′, 10′-Oxygenase Gene (EcBCO2) From *Exopalaemon Carinicauda*. *International Journal of Biological Macromolecules*.

[47] Wu L., Guo X., Wang W. (2016). Molecular Aspects of B, B-Carotene-9′, 10′-Oxygenase 2 in Carotenoid Metabolism and Diseases. *Experimental Biology and Medicine*.

[48] Amengual J., Lobo G. P., Golczak M., Hua N. M. L., Lintig J. V. (2010). A Mitochondrial Enzyme Degrades Carotenoids and Protects Against Oxidative Stress. *The FASEB Journal*.

[49] Mein J. R., Dolnikowski G. G., Ernst H., Russell R. M., Wang X.-D. (2011). Enzymatic Formation of Apo-Carotenoids From the Xanthophyll Carotenoids Lutein, Zeaxanthin and B-Cryptoxanthin by Ferret Carotene-9′,10′-Monooxygenase. *Archives of Biochemistry and Biophysics*.

[50] Zhang Y., Jin J., Wang N. (2024). Cytochrome P450 CitCYP97B Modulates Carotenoid Accumulation Diversity by Hydroxylating B-Cryptoxanthin in *Citrus*. *Plant Communications*.

[51] Mundy N., Stapley J., Bennison C. (2016). Red Carotenoid Coloration in the Zebra Finch Is Controlled by a Cytochrome P450 Gene Cluster. *Current Biology*.

[52] Xu Z., Liang Q., Chen Z., Dong Z., Guo Y., Wang Z. (2023). Weighted Gene Co-Expression Network Analysis of Red Body Color Formation of Crimson Snapper, *Lutjanus Erythropterus*. *Aquaculture Reports*.

[53] Goncalves M. B. C. V., Agudo M., Connor S. (2009). Sequential RARβ and A Signalling, *In Vivo*, Can Induce Adult Forebrain Neural Progenitor Cells to Differentiate Into Neurons Through Shh and FGF Signalling Pathways. *Developmental Biology*.

[54] Catherino W. H., Malik M. (2007). Uterine Leiomyomas Express a Molecular Pattern That Lowers Retinoic Acid Exposure. *Fertility and Sterility*.

[55] Chang C. L., Hong E., Lao-Sirieix P., Fitzgerald R. C. (2008). A Novel Role for the Retinoic Acid-Catabolizing Enzyme Cyp26a1 in Barrett’s Associated Adenocarcinoma. *Oncogene*.

[56] Ross A. C., Zolfaghari R. (2011). Cytochrome P450s in the Regulation of Cellular Retinoic Acid Metabolism. *Annual Review of Nutrition*.

[57] Fernandes S. S., Arcuri R., Morgado-Díaz J. A., Benchimol M. (2004). Increase of Melanogenesis by Retinoic Acid: An Ultrastructural and Morphometric Study. *Tissue and Cell*.

[58] Yu H. R., Chen H. P., Wang X. X. (2024). Sws2 Gene Positively Regulates Melanin Production in *Plectropomus Leopardus* Skin via Direct Regulation of the Synthesis of Retinoic Acid. *International Journal of Molecular Sciences*.

[59] Chatzifotis S., Pavlidis M., Jimeno C. D., Vardanis G., Sterioti A., Divanach P. (2005). The Effect of Different Carotenoid Sources on Skin Coloration of Cultured Red Porgy *Pagrus Pagrus*. *Aquaculture Research*.

[60] Wu M., Chen X., Cui K., Li H., Jiang Y. (2020). Pigmentation Formation and Expression Analysis of Tyrosinase in *Siniperca Chuatsi*. *Fish Physiology and Biochemistry*.

[61] Hsu C.-H., Liou G.-G., Jiang Y.-J. (2020). Nicastrin Deficiency Induces Tyrosinase-Dependent Depigmentation and Skin Inflammation. *Journal of Investigative Dermatology*.

[62] Naikoo S. H., Rashid H., Kumarm S. (2023). Inhibition of Melanogenesis by 3-(1′-Methyltetrahydropyridinyl)-2,4-6-Trihydroxy Acetophenone Via Suppressing the Activity of Camp Response Element-Binding Protein (Creb) and Nuclear Exclusion of Creb-Regulated Transcription Coactivator 1 (Crtc1). *European Journal of Pharmacology*.

[63] Kim D. H., Shin D. W., Lim B. O. (2023). Fermented Aronia melanocarpa Inhibits Melanogenesis Through Dual Mechanisms of the PI3K/AKT/GSK-3β and PKA/CREB Pathways. *Molecules*.

[64] Wu K. C., Hseu Y. C., Shih Y. C. (2022). Calycosin, A Common Dietary Isoflavonoid, Suppresses Melanogenesis Through the Downregulation of PHA/CREB and P38 MAPK Signaling Pathways. *International Journal of Molecular Sciences*.

[65] Hoekstra H. E. (2006). Genetics, Development and Evolution of Adaptive Pigmentation in Vertebrates. *Heredity*.

[66] Agulleiro M. J., Cortes P., Leal E., Rios D., Sanchez E., Cerda-Reverter J. M. (2014). Characterization, Tissue Distribution and Regulation by Fasting of the Agouti Family of Peptides in the Sea Bass *Dicentrarchus Labrax*. *General and Comparative Endocrinology*.

[67] García-Borrón J. C., Sánchez-Laorden B. L., Jiménez-Cervantes C. (2005). Melanocortin-1 Receptor Structure and Functional Regulation. *Pigment Cell Research*.

[68] Shamsi A., Khan M. S., Altwaijry N., Hassan N., Shahwan M., Yadav D. K. (2025). Targeting PDE4A for Therapeutic Potential: Exploiting Drug Repurposing Approach Through Virtual Screening and Molecular Dynamics. *Journal of Biomolecular Structure and Dynamics*.

[69] Schmetterer K. G., Goldhahn K., Ziegler L. S., Gerner M. C., Marculescu R. (2019). Overexpression of PDE4A Acts as Checkpoint Inhibitor Against Camp-Mediated Immunosuppression *In Vitro*. *Frontiers in Immunology*.

[70] Sachs B. D., Akassoglou K. (2007). Regulation of Camp by the P75 Neurotrophin Receptor: Insight Into Drug Design of Selective Phosphodiesterase Inhibitors. *Biochemical Society Transactions*.

[71] Zhou S. Q., Sakamoto K. (2020). Citric Acid Promoted Melanin Synthesis in B16F10 Mouse Melanoma Cells, but Inhibited It in Human Epidermal Melanocytes and HMV-II Melanoma Cells Via the GSK3β/B-Catenin Signaling Pathway. *PLOS ONE*.

[72] Lee C.-L., Gao Z.-A., Jhan Y.-L., Chang Y.-S., Chen C.-J. (2021). Tuliposides H-J and Bioactive Components from the Bulb of *Amana Edulis*. *Molecules*.

[73] Gunia-Krzyzak A., Popiol J., Marona N. (2016). Melanogenesis Inhibitors: Strategies for Searching for and Evaluation of Active Compounds. *Current Medicinal Chemistry*.

[74] Zhong L., He Q., Xu M., Chen F.-F., Li F., Chen Y.-P. (2024). Unveiling *Acetobacter Syzygii* From Tibetan Kefir Grain: Fermentation-Enhanced Anti-Tyrosinase, and Anti-Melanin. *Fermentation-Basel*.

[75] Slominski A., Paus R. (1994). Towards Defining Receptors for L-Tyrosine and L-Dopa. *Molecular and Cellular Endocrinology*.

[76] Schwahn D. J., Xu W. D., Herrin A. B., Bales E. S., Medrano E. E. (2001). Tyrosine Levels Regulate the Melanogenic Response to A-Melanocyte-Stimulating Hormone in Human Melanocytes: Implications for Pigmentation and Proliferation. *Pigment Cell Research*.

[77] Zhang S. Y., Wang T. W., Huang C. W. (2025). *Effects of Marigold*, *Astaxanthin and Amaranth on the Pigmentation of Large Yellow Croaker* (*Larimichthys Crocea*).

